# Range-Finding Risk Assessment of Inhalation Exposure to Nanodiamonds in a Laboratory Environment

**DOI:** 10.3390/ijerph110505382

**Published:** 2014-05-16

**Authors:** Antti J. Koivisto, Jaana E. Palomäki, Anna-Kaisa Viitanen, Kirsi M. Siivola, Ismo K. Koponen, Mingzhou Yu, Tomi S. Kanerva, Hannu Norppa, Harri T. Alenius, Tareq Hussein, Kai M. Savolainen, Kaarle J. Hämeri

**Affiliations:** 1 Nanosafety Research Centre, Finnish Institute of Occupational Health, Topeliuksenkatu 41 a A, FI-00250 Helsinki, Finland; E-Mails: jaana.palomaki@tukes.fi (J.E.P.); anna-kaisa.viitanen@ttl.fi (A.-K.V.); kirsi.siivola@ttl.fi (K.M.S.); tomi.kanerva@ttl.fi (T.S.K.); hannu.norppa@ttl.fi (H.N.); harri.alenius@ttl.fi (H.T.A.); kai.savolainen@ttl.fi (K.M.S.); 2 National Research Centre for the Working Environment, Lersø Parkallé 105, Copenhagen DK-2100, Denmark; E-Mail: ikk@arbejdsmiljoforskning.dk; 3 Institute of Earth Environment, Chinese Academy of Sciences, Fenghui Road, Xi’an 710075, China; E-Mail: yumingzhou1738@yahoo.com; 4 Department of Physics, Faculty of Science, The University of Jordan, Amman JO-11942, Jordan; 5 Department of Physics, University of Helsinki, Gustaf Hällströmin Katu 2, P.O. Box 64, Helsinki FI-00014, Finland; E-Mails: tareq.hussein@helsinki.fi (T.H.); kaarle.hameri@helsinki.fi (K.J.H.)

**Keywords:** nanomaterial, occupational hygiene, source characterization, exposure assessment, dose assessment, *in vitro*

## Abstract

This study considers fundamental methods in occupational risk assessment of exposure to airborne engineered nanomaterials. We discuss characterization of particle emissions, exposure assessment, hazard assessment with *in vitro* studies, and risk range characterization using calculated inhaled doses and dose-response translated to humans from *in vitro* studies. Here, the methods were utilized to assess workers’ risk range of inhalation exposure to nanodiamonds (NDs) during handling and sieving of ND powder. NDs were agglomerated to over 500 nm particles, and mean exposure levels of different work tasks varied from 0.24 to 4.96 µg·m^−3^ (0.08 to 0.74 cm^−3^). *In vitro*-experiments suggested that ND exposure may cause a risk for activation of inflammatory cascade. However, risk range characterization based on *in vitro* dose-response was not performed because accurate assessment of delivered (settled) dose on the cells was not possible. Comparison of ND exposure with common pollutants revealed that ND exposure was below 5 μg·m^−3^, which is one of the proposed exposure limits for diesel particulate matter, and the workers’ calculated dose of NDs during the measurement day was 74 ng which corresponded to 0.02% of the modeled daily (24 h) dose of submicrometer urban air particles.

## 1. Introduction

Industrial use of materials engineered at the nanometer scale has grown rapidly worldwide [1]. Nanotechnology has been identified as one of the most promising technologies in advanced societies [2]. In Europe alone, the nanotechnology sector is believed to employ 400,000 persons by 2015 [3]. However, during the last decade, there has been a growing amount of evidence that some engineered nanomaterials (ENMs; see definition by ISO [4]) may be toxic to humans (e.g., [5,6,7]).

**Figure 1:**
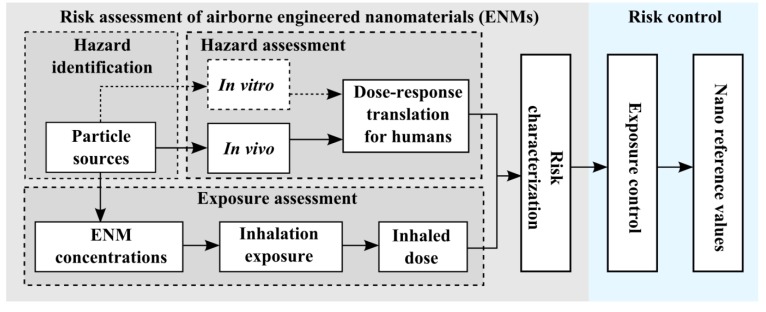
Principal steps of ENM risk assessment: identify particle sources, assess ENM hazard and exposure levels, and characterize risk of the ENM exposure. Exposure control measures are based on the risk level which can be used to estimate an upper limit on the acceptable ENM concentration in workplace air.

Exposures to ENMs most readily occur when the materials are produced and handled [5,8]. For such exposure, inhalation is the most relevant pathway for ENM uptake [9]. Quantitative risk assessment and control of ENMs is currently based on ENM exposure assessment and hazard assessment with *in vivo* studies (Figure 1). 

Currently, there are no health-based exposure limits or toxicological test guidelines specific for ENMs. This is because the use of conventional protocols [10] to derive no-effect levels is challenging to use for particulate matter due to the lack of adequate data. Thus, the risk assessment of exposure to ENMs currently relies on hazard assessment based on available toxicological data [11].

Risk assessment of airborne ENMs (Figure 1) begins with identifying particle emitters [12], where size resolved ENM concentrations are discriminated from background particles [13,14,15], and the ENM exposure levels are used to calculate inhaled doses [15,16]. The ENM hazards are assessed with *in vivo* experiments for specific toxicological endpoints [17,18] and translated to the human equivalent dose-response [10,19]. Such risk assessment has been suggested for workers exposed to TiO_2_ nanoparticles [20,21,22,23] and carbon black particles during material production [21]. However, some of these risk assessment models have been criticized mainly due to inappropriate dose–response assessment and exposure data [24,25,26].

The scientific and regulatory communities are currently discussing ways to develop faster and more predictive tools for assessing ENM hazards such as using *in vitro* experiments [27,28,29,30]. Snyder-Talkington *et al.* [31] used *in vitro* studies to evaluate hazards of multi-walled carbon nanotubes which were linked via mouse equivalent dose to the human equivalent dose. They postulated that a 10 μg dose given to a mouse represents a (nominal) concentration of less than 1 μg·mL^−1^ *in vitro* corresponding to a human equivalent inhalation dose where a worker is exposed to an aerosol concentration of 400 μg·m^−3^ for one month. Recently Hinderliter *et al.* [32] developed an *in vitro* cell dosimetry model that may be used to accurately estimate the delivered (settled) dose onto cells if proper ENM dispersion protocol is used [33].

This study considers the risk assessment of inhalation exposure to ENMs (Figure 1), applied to workers handling Molto^®^ nanodiamond (ND) powder (see material data sheet by vendor in Supplementary Information Figure S1 and Mochalin *et al.* [34] for ND synthesis, properties, and use). Measured particle size distributions were used to discriminate the ND concentrations from background particles and ND emission rates were characterized during handling, sieving, and cleaning of NDs. Work task based mean ND exposure levels were used to calculate workers inhaled dose rates. The cytotoxicity of NDs was studied in the THP-1 cell line by assessing cell death and ability to produce reactive oxygen species (ROS) and IL-1β, IL-8, and TNF-α cytokines. Assessment of the *in vitro* dose-deposited on cells was not considered reliable, due to the fact that the model did not take into account ND agglomeration in dispersion, and thus risk range characterization was not performed. The ND exposure and dose levels were compared with one of the proposed exposure limits for diesel particulate matter and with the inhaled dose of urban particulate matter exposure.

## 2. Experimental Section

### 2.1. Work Environment

**Figure 2:**
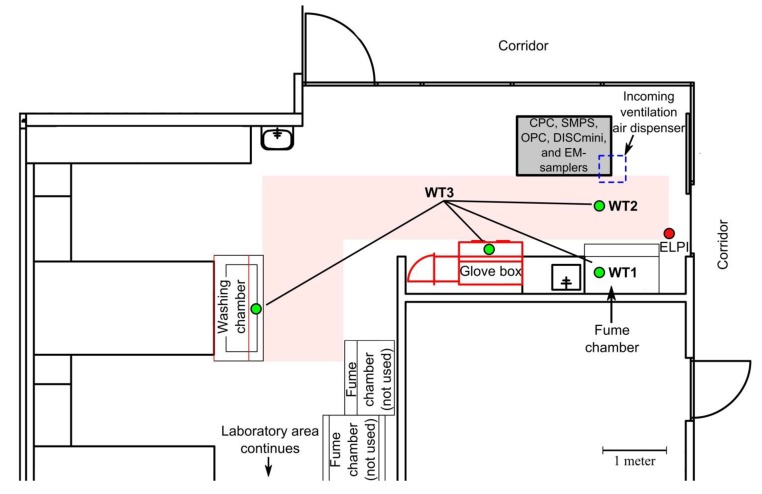
Layout of the work area. Light red shows the ND handling area during work tasks.

The workplace was located in a sub-urban area in a modern office building. The incoming ventilation air was filtered with a EU7 grade filter (HGFS-F7-592/892/600-8-25, Camfil Farr, Stockholm, Sweden), and during working hours, the ventilation rate was set to approximately 2 h^−1^. In the laboratory, daytime relative humidity and temperature were, on average, 20% and 23 °C, respectively. During work tasks, the laboratory doors were closed and the room was at a 6 Pa under pressure compared to the corridor (Figure 2). ND sieving was performed with the vibratory sieve shaker (Retsch AS 300, Retsch Technology GmbH, Haan, Germany).

Figure 2 shows the layout of the laboratory, location of the instruments, and area where NDs were handed. There were three work tasks: (a) ND handling in a glove box and sieving in a fume chamber (**WT1**); (b) ND handling in a under pressurized glove box and sieving in a room (**WT2**); and (c) cleaning operations (**WT3**). During the work day, 2 kilograms of ND powder was handled. In the glove box, ND bags were opened, weighed and poured on the sieve. The closed sieve containing NDs from 200 to 500 grams was transported to the sieve by hands where it was opened and attached to the vibratory shaker. Duration of the sieving varied from a few minutes to ten minutes. In cleaning operations, surfaces were wiped with wet tissues, components were washed in a washing chamber, and empty ND bags were disposed of into special waste bins. During the work tasks, workers wore face piece respirators (Willson 5321 FFP3D, W1005602, Bacou-Dalloz USA, Smithfield, RI, USA) of which the nominal protection factors were 200 (European standard EN529 [35]), cotton clothing, nitrile gloves, and sleeve protectors.

### 2.2. Aerosol Measurements

The particle number concentration was measured from a workstation with a condensation particle counter (CPC, *Q_s_* = 0.70 L·min^−1^, TSI Model 3007, TSI Inc., Shoreview, NM, USA). Measurement size range was approximately from 10 nm to over 1 µm with a time resolution of 1 s.

The breathing zone concentration was measured with a Miniature Diffusion Size Classifier (DISCmini, *Q_s_* = 0.96 L·min^−1^, sampling through 1 m with inner diameter of 4 mm conductive rubber tube without a cyclone or pre-impactor and the inlet located approximately 15 cm from the worker mouth). DISCmini is a diffusion charger that measures the current carried by the aerosol particles [36,37]. The current is proportional to particle diameter from *D_p_*^1^ to *D_p_*^2^, which is referring to the active surface area of particulate matter [38,39,40]. The measurement size range was approximately from 20 to ~700 nm and the instrument time resolution was 1 s.

The work station air and incoming ventilation air particle concentrations were measured with a scanning mobility particle sizer (SMPS), which consisted of a classifier (TSI Classifier model 3080) and a 10 μCi 85Kr neutralizer, TSI model 3077A), a HAUKE-type differential mobility analyzer (DMA, length 28 cm, inner diameter 2.5 cm and outer diameter 3.3 cm, sheath air flow 6.0 L·min^−1^, and a condensation particle counter (CPC, TSI model 3776, *Q_s_* = 1.43 L·min^−1^). The SMPS scan time was 45 s with a 15 s retrace time. The aerosol was sampled with a 3-way valve altering between the work station (length 1.2 m, tube inner diameter 4 mm) and the incoming ventilation air dispenser (length 4 m, tube inner diameter 4 mm). Both sampling lines were flushed with flows of 3.6 L min^−1^ to decrease the sample residence time. Diffusion losses of the sampling lines were corrected according to Baron and Willeke [41]. The measurement size range of the SMPS was from 14.1 nm to 572.5 nm.

An optical particle counter (OPC, *Q_s_* = 1.18 L·min^−1^, and the instrument default settings were: refractive index 1.59 + 0i, density 2.6 g·cm^−3^, and spherical particle assumption, Model 1.109, Grimm Aerosoltechnik, Ainring, Germany) measured work station optical particle mass size distributions from 234.5 nm to 31 μm with a time resolution of 60 s. These particle mass size distributions were transformed to particle number and mass distributions for which the density was assumed to be 1 g·cm^−3^ for particles below 500 nm in diameter and 0.5 g·cm^−1^ for particles over 500 nm in diameter. This corresponds to the ND powder bulk density (see Supplementary Information) and roughly the effective density of atmospheric particles in urban area with a size greater than 500 nm in diameter [42].

The OPC size distributions were averaged to two minute samples that corresponded to the SMPS work station measurement time interval. Then, the work station size distributions measured by the SMPS and OPC were combined by removing the OPC size fractions smaller than 289.2 nm (lower boundary 278.4 nm) and the SMPS size fractions of over 278.8 nm. The SMPS largest size bin was cut 0.4 nm so that the upper boundary limit was the same as the OPC smallest size bin lower boundary limit of 278.4 nm. For the SMPS cut size bin was calculated new geometric mean size d50, bin concentration d*N*, and logarithmic width of the bin dLog(*D_p_*). Thus, the combined size distribution named here as SMPS + OPC, was continuous and ranged from 14.1 nm to 31 μm consisting of 111 size classes. Here it was assumed that a particle’s mobility diameter and optical diameter were equivalent even though optical diameter may differ significantly from mobility diameter depending on the particles refractive index and shape [43,44].

Particle aerodynamic size distributions were measured from the work station with an electrical low-pressure impactor with a filter stage (ELPI, *Q_s_* = 9.6 L·min^−1^, Dekati Ltd., Tampere, Finland). Measurement time resolution was 1 s, which was averaged for data analysis to 60 s. The measurement size range was 7 nm to 10 µm (13 size classes), and in the data inversion, the density was assumed to be 1 g cm^−3^. Room temperature and relative humidity were measured with a TR-73U logger (Thermo, T&D Corporation, Nagano, Japan).

The instruments were placed on a measurement trolley where the inlets were at a height from 1.2 to 1.4 m facing towards the work area (Figure 2). Measurements were taken over three consecutive days where the first and last days were used to set up the instrumentation and for non-working hour concentration measurements. The actual work was performed during the second day between 9:31 and 15:23.

### 2.3. Electron Microscopy

Electron microscope samples were collected with a Nanoguard (IUTA/TSI) electrostatic precipitator pre-prototype on Si substrate and onto holey carbon film-coated copper grids of 200-mesh (SPI, West Chester, PA, USA) to air sample cassettes (inlet diameter 1/8 inch and filter diameter 25 mm, SKC Inc., 863 Valley View Road Eighty Four, PA, USA) on polycarbonate filter. Samplers located at the measurement trolley. Si substrate was imaged by using an UltraPLUS FEG-SEM (Zeiss, Oberkochen, Germany) and INCA Energy 350 EDS-system (Oxford Instruments NanoAnalysis, High Wycombe Bucks, England) and grids by using a transmission electron microscope (TEM, JEM 1220, Jeol, Tokyo, Japan).

### 2.4. Dynamics of Nano-diamonds: Emissions and Settling

The dynamic behavior of aerosol particles can be described by the general mass-balance equation [12]. Here, it was applied to describe the mass concentration change, d*M*/d*t* (µg·s^−1^·m^−3^), at the work station as:

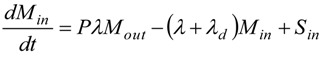
(1)
where *M* (µg·m^−3^) is the mass concentration with in and out referring to the indoor and outdoor concentration respectively, *P* (unitless) is the penetration factor of aerosols while being transported from the outdoor into the indoor air, *λ* (h^−1^) is the ventilation rate, *λ_d_* (h^−1^) is the deposition rate of aerosols, and *S_in_* (µg·s^−1^·m^−3^) is the source rate of producing aerosols indoors (mass-based).

Assuming that the indoor aerosol particles are produced with significantly large amounts so that the term *S_in_* is much larger than other two terms on the right hand side, then the source strength can be easily calculated as:

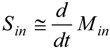
(2)

When the process emission stops, mass concentration decay to the background concentration level after a time period that depends on the ventilation rate and the deposition rate; *i.e.*, the deposition rate of aerosols right after the source was terminated, can be estimated as:

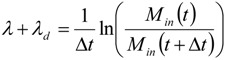
(3)
where Δ*t* is the time interval for the indoor aerosol mass concentration to decay at the background concentration level.

### 2.5. Calculation of Regional Inhalation Dose

The regional inhalation dose rate (min^−1^) was calculated by multiplying size fractioned concentrations by the respiratory minute volume (L·min^−1^) and the regional deposition probabilities while air is inspired and expired. In this model, we used the human respiratory tract deposition probability model as determined by the International Commission on Radiological Protection [45]. 

Deposition probabilities are defined as a function of aerodynamic diameter, which is linked to a mobility diameter with an effective density [46]. In this study, we assumed that particle densities would be 1 g·cm^−3^ for particles below 500 nm in diameter and 0.5 g·cm^−1^ for particles over 500 nm in diameter. For the lung deposition calculation over 500 nm particles size was converted to aerodynamic equivalent diameter according to Hinds [47] where particles dynamic shape factor was set to 1.37. This is typical value for compact agglomerates for which the dynamic shape factor varies usually from 1.3 to 1.4 [48]. Particle growth during inspiration and expiration was assumed to be negligible and workstation concentrations were assumed to correspond to the workers exposure concentrations. The workers’ respiratory minute volume was assumed to be 25 L·min^−1^, which corresponds to male respiration during light exercise. The regional deposition probabilities were calculated by using simplified regional deposition probabilities during nasal breathing for the head airways, the tracheobronchial region, and the alveolar region [49]. 

### 2.6. In vitro-experiments

#### 2.6.1. Cell Culture

The human monocytic leukaemia cell line THP-1 was obtained from the American Type Culture Collection (cat. TIB-202, ATCC, Rockville, MD, USA) and maintained in RPMI 1640 cell culture medium (Invitrogen, Paisley, UK) supplemented with 1% L-glutamine (Ultraglutamine^®^; Lonza, Basel, Switzerland), 10% fetal bovine serum (FBS, Gibco by Life Technologies, Paisley, UK), 1% HEPES (Lonza), 1% penicillin-streptomycin (PEST; Invitrogen), 1% non-essential amino acids (Lonza) and 0.05 mM 2-mercaptoethanol (Lonza). In order to induce monocyte-to-macrophage differentiation, the THP-1 cells were cultured for 48 h in the standard culture medium supplemented with 100 nM phorbol 12-myristate 13-acetate (PMA; Sigma-Aldrich, Schnelldoff, Germany). The cells were seeded into 12-well plates (Multiwell™ TC Plates, Cat. No. 35304, BD Biosciences Discovery Labware, Franklin Lakes, NJ, USA) at a density of 5 × 10^5^/well to study cell death and cytokine secretion and into 24-well plates (Wallac Visiplate, Visiplate-24, PerkinElmer, Zaventem, Belgium) at a density of 3 × 10^5^/well to study ROS generating ability. 

#### 2.6.2. Dispersion Preparation

Dispersions of Molto^®^ ND for the *in vitro* experiments were made up by weighing NDs into glass tubes and preparing a 1,000 μg·mL^−1^ stock dispersion in phosphate buffered saline (dPBS) which was sonicated for 20 min at 30 °C. The stock solution was further serially diluted to form different final nominal concentrations in RPMI 1640 cell culture media with supplemental 1% PEST and 1% L-glutamine (LDH and ELISA assays) or Dulbecco’s phosphate-buffered saline (dPBS without CaCl_2_/MgCl_2_, Gibco by Life Technologies; ROS generating ability) and sonicated for 20 min at 30 °C just before cell exposures. Old media was carefully removed and the cells were exposed to different doses of NDs for 3 h (ROS generating ability) or 6 h (LDH, ELISA assays).

#### 2.6.3. Analysis of Cell Death and Cytokine Secretion

The ability of Molto^®^ ND to evoke cell death was investigated via the release of lactase dehydrogenase enzyme (LDH) into the cell culture medium, by the cytotoxicity detection kitPLUS (Roche, Mannheim, Germany) according to manufacturer’s instructions. The ND nominal concentrations used in the cytotoxicity tests were 1, 10, 100 and 500 μg·mL^−1^. The secretion of pro-inflammatory cytokines IL-1β, TNFα and IL-8 was studied by commercial enzyme-linked immunosorbent (ELISA) assays after the THP-1 cells had been exposed to NDs at 10 and 100 μg·mL^−1^. All ELISA assays were purchased from eBiosciences (San Diego, CA, USA), and performed according to the manufacturer’s instructions. Bacterial lipopolysaccharide *Escherichia coli* serotype O111:B4 (LPS, Sigma-Aldrich) was used as a positive control because of its known ability to activate the secretion of pro-inflammatory cytokines from macrophages. Two independent repeats of the experiments were performed.

#### 2.6.4. Determination of ROS Generating Ability

The effect of Molto^®^ ND on ROS generation was assessed both with THP-1 cells and in the cell-free environment. 2',7'-Dichlorofluorescein diacetate (DCFH-DA, Calbiochem, Darmstadt, Germany) was used to detect intracellular ROS production. After DCFH-DA has crossed cell membrane, it is de-esterified to a non-fluorescent 2',7'-dichloridihydrofluorescein (DCFH) which is unable to pass back through cell membranes and therefore it cannot leave the cells. DCFH can be oxidised to the intracellular, fluorescent 2',7'-dichlorofluorescein (DFC) by reactive oxygen species, such as hydroxyl radicals, peroxynitrite, and nitric oxide [50,51]. In the cell free environment, DCFH-DA can be chemically hydrolysed to DCFH by treatment with 0.01 M NaOH for 30 min, after which the test material is added and its ROS producing capability is determined [50]. 

THP-1 cells were seeded into 24-well plates (Wallac Visiplate, Perkin Elmer) and differentiated into macrophages as described above. The DCFH-DA solution was diluted into dPBS, THP-1 cells were washed twice with dPBS, and DCFH-DA was added to the cells at 0.01 mM final concentration. After 1 h, the cells were washed once with dPBS and exposed for 3 h to 10, 100 and 500 μg·mL^-1^ ND dispersions prepared as described above. Three independent repeats of the cell experiments were performed. In addition, parallel experiments using DCFH were performed in the cell-free environment with appropriate unlabelled controls being included. DFC was determined at *λ_ex_* 490 and *λ_em_* 520 nm on a fluorescence spectrophotometer (Victor Wallac-2 1420, Perkin Elmer). H_2_O_2_ (500 mM) was used as a positive control. The results are given as intracellular H_2_O_2_ concentrations (nM) calculated with the following equation: H_2_O_2_ (nM) = 0.0004 × fluorescence-86.

## 3. Results

**Figure 3:**
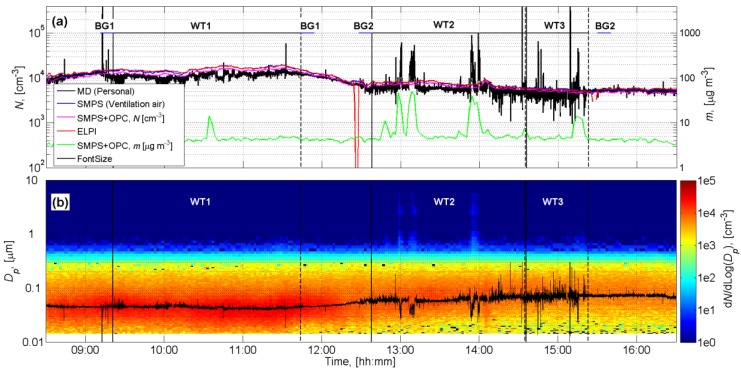
Concentration time series during the work day: (**a**) shows the particle and mass concentrations; (**b**) shows the particle size distributions measured with the SMPS + OPC and the average particle size defined by the DISCmini. The horizontal blue lines and the horizontal black lines in (**a**) show the start and end time for background concentrations (BG1 and BG2) and work tasks (**WT1**, **WT2**, and **WT3**), respectively. The vertical solid and dashed thin black lines show the start and end times of the work tasks.

Figure 3a,b show the particle concentration time series measured during the work day between 8:30–16:30. The work station air concentration measured by the SMPS+OPC before and after the work day between 16:30–8:30 were on average 4,600 ± 2,900 particles cm^−3^ and 3.05 ± 1.27 μg·m^−3^ (± refers to one standard deviation).

**Figure 4:**
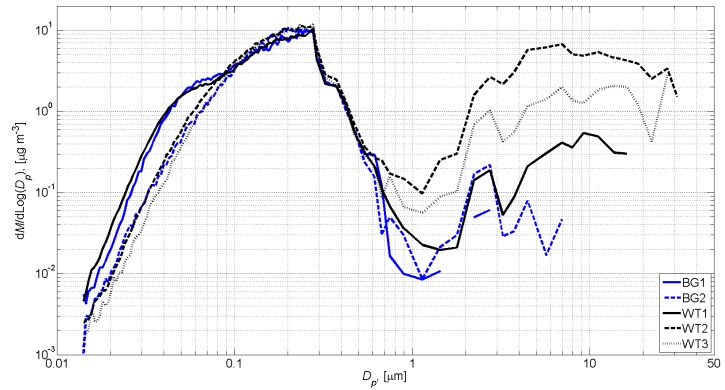
Mass distribution averages for the background’s (BG1 and BG2) and work task’s (**WT1**, **WT2**, and **WT3**) concentrations calculated from the SMPS + OPC measurements.

The average background concentrations were defined before and after work tasks as follows: (a) background concentration 1 (BG1) measured from the work station air between 9:11–9:21 and 11:44–11:54; and (b) background concentration 2 (BG2) measured from the work station air between 12:28–12:38 and 15:30–15:40 (Figure 3a). BG1 and BG2 were defined separately for each instrument. The work task concentrations were averaged over the measurement period for each instrument as follows: **WT1** 09:21–11:44, **WT2** 12:38–14:35, and **WT3** 14:36–15:23 (Figure 3a). The **WT2** and **WT3** concentrations had the same background concentration (BG2). Mass distribution averages for the background’s and work task’s concentrations are shown in Figure 4.

**Figure 5:**
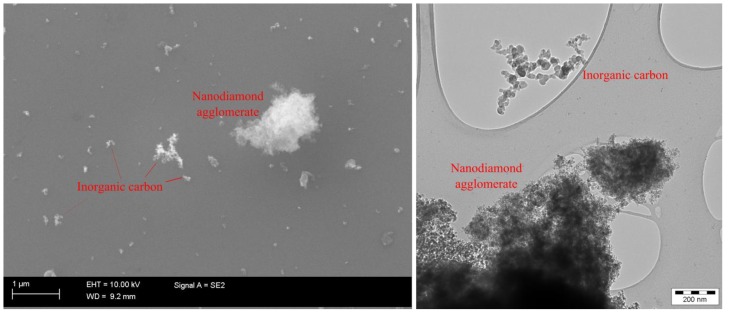
Scanning (left) and transmission (right) electron micrographs of particles sampled from the work station air.

Electron microscopy analysis showed that the work station airborne particles consisted of inorganic carbon (data not shown), which originated from outdoor combustion sources, agglomerated NDs, and other particles whose composition was not defined (Figure 5).

### 3.1. Exposure Assessment

The Appendix shows that the most reliable metrics to characterize ND exposure levels was mass concentration derived from the SMPS + OPS measurements. Mass emission rates of ND agglomerates were estimated for six major mass concentration peaks shown in Figure 3a. In the emission rate modelings, ND agglomerates were estimated to have a mean diameter of 5 μm. The deposition rate was found to vary from 0.8 to 1.9 h^−1^. The variation was most likely caused by incomplete air mixing and air mass movement caused by the workers’. The maximum ND emission rates for **WT1**, **WT2**, and **WT3** were respectively 6.1, 12.4, and 2.2 μg·min^−1^·m^−3^, while particles were emitted to the work station air at a rate of 4.3 μg·min^−1^·m^−3^ when ND emissions were excluded (Table 1). It must be noted that Equation (1) assumes particles fully mixed in air which may not be valid in this case. Thus the emission rate term was expressed as (units·min^−1^·m^−3^). The air mixing without workers can be estimated with computational fluid dynamics.

Figure 3a shows that the breathing zone concentration measured by the DISKmini and work station mass concentration peaks occurred approximately at the same time. This supports the assumption that the work station air concentrations and the exposure levels were similar. The work tasks’ mean ND particle number and mass size distributions were calculated by subtracting BG1 from **WT1** and BG2 from **WT2** and **WT3** (Figure 4), and excluding particles below 500 nm in diameter. These concentrations were used to calculate work task based ND dose rates and doses where workers’ use of the respirators with nominal protection factor of 200 was taken into account (Table 1).

**Table 1:** ND exposures, emission rates for mass peak concentrations during work tasks, and calculated dose rates, and doses of ND agglomerates to the head-airways (H-A), trachea-bronchiolar (TB), and alveolar regions. The exposure concentration was defined from the SMPS + OPC particle size distributions where particles smaller than 500 nm in diameter were removed. Mass concentration was calculated by assuming that the particles effective density was 0.5 g·cm^−3^. The dose was calculated over the time period of respective work task.

Unit	WT1		WT2		WT3	
ND exposure **^a^**	0.08 cm^−3^	0.24 μg m^−3^	0.74 cm^−3^	4.96 μg·m^−3^	0.30 cm^−3^	1.54 μg·m^−3^
ND emission rate	6.1 μg·min^−1^·m^−3^		1.7 to 12.4 μg·min^−1^·m^−3^		2.2 μg·min^−1^·m^−3^	
Dose rate **^b^**	2.2 min^−1^	0.03 ng·min^−1^	26.0 min^−1^	0.53 ng·min^−1^	8.8 min^−1^	0.16 ng·min^−1^
Dose **^c^**	310	4 ng	3,100	62 ng	410	8 ng
H-A, (%)	59	87	66	88	62	90
TB, (%)	5	5	6	5	5	4
Alveolar, (%)	36	8	28	7	33	6

Notes: **^a^** ND concentration in workstation air; **^b,c^** The use by workers of respiratory protection with a nominal protection factor of 200 was taken into account.

### 3.2. Assessment of NDs Toxicity in vitro

In order to study the mass based dose-response of NDs on cells, THP-1 cells were exposed to 1, 10, 100 and 500 μg·mL^−1^ ND dispersions. The ND agglomerates growth in the dispersion prevented to use the *in vitro* dosimetry model [32] to calculate the dose delivered on the THP-1 cells. The ND dispersion characteristics, *in vitro* dose-responses, and dose translation from *in vitro* to humans is presented in Supplementary Information, but the results were not used in this study.

**Figure 6:**
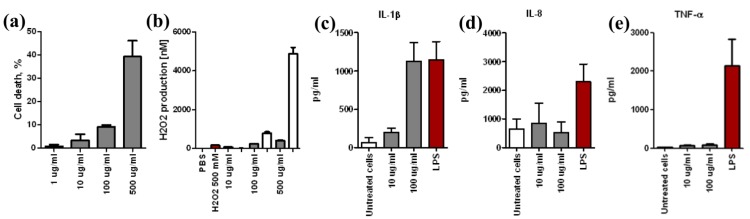
Nanodiamond Molto particles induce pro-inflammatory response. (**a**) Cell death of macrophages induced by 1, 10, 100 and 500 μg/mL of NDs; (**b**) PBS, H_2_O_2_ positive control with cells, Acellular (open bars), and cellular (filled bars) ROS production induced by 10, 100 and 500 μg/mL of NDs; (**c**) IL-1β; (**d**) IL-8 and (**e**) TNF-α cytokine secretion from macrophages induced by 10 and 100 μg/mL of NDs. Error bars represent ±SD of two (**a**, **c**–**e**) or three (**b**) independent experiments.

Cytotoxicity depended on ND nominal concentration as seen in Figure 6a. The NDs showed a clear capability to trigger ROS production in the cell-free environment. The intracellular production of ROS was lower, although it depended on the nominal mass concentration of ND particles (Figure 6b). The levels of secreted IL-1β protein were increased dose-dependently, whereas there were no changes in TNF-α and IL-8 protein secretion compared to untreated cells (Figure 6c–e). The results suggest that exposure to nano-sized ND evoked biological responses in the exposed cells.

## 4. Discussion

NDs agglomerated to over 500 nm particles and their levels in terms of number and surface area concentration was low as compared to background levels (see Appendix). Mass units were used for the ND exposure assessment because of reliability (see Appendix) and because a toxicologically relevant metric is not defined for ND agglomerates. It must be noted that single ND agglomerate carries millions of primary ND particles 5–6 nm in diameter (Figure 2, Supplementary Information Figure S1) which may be dispersed when deposited on the respiratory fluid. Choi *et al.* [52] showed that nanoparticles with *d_H_* < 34 nm translocate rapidly from the lungs to lymph nodes. From lymph nodes, nanoparticles with *d_H_* < 6 nm moved to the bloodstream and extrapulmonary organs. Exposure to urban air nano-sized particles has been found to induce health effects such as cardiovascular [53] and central nervous diseases [54].

Even though the ND agglomerates are mainly deposited in the head airways, it was calculated that 7% of them penetrated to the alveolar region. In alveoli, macrophages are the first cells to encounter the particles as they act as the first-line of defense cells and engulf invading particles [55]. They also orchestrate the inflammatory response by secreting cytokines and chemokines, which recruit other inflammatory cells into the lungs. This cross-talk between different cell types is an important part of the pro-inflammatory response and macrophages represent the first step to trigger the activation cascade of the inflammatory response [31].

Nanodiamond Molto^®^ showed relatively low cytotoxicity, a marked production of ROS, and a dose-dependent secretion of the pro-inflammatory cytokine, IL-1β, in THP-1 cells. The production of intracellular ROS has been shown to be associated with the secretion of IL-1β via NLRP3-inflammasome activation orchestrating the activation of the pro-inflammatory cascade in cells exposed to cholesterol or silica crystals, crocidolite asbestos or rigid carbon nanotubes [56,57,58]. Activation of this pathway has been suggested to be an important tool for separating hazardous nano-sized fibers from their non-hazardous counterparts [58]. With spherical shaped ENM, the suitability of NLRP3 inflammasome activation studies to the hazard identification process is still unclear. Thus the role of the NLRP3 inflammasome in fiber and particle induced inflammation has to be further studied in the future.

To estimate the dose delivered (settled) on the cells with the *in vitro* dosimetry model by Hiderliter *et al.* [32], a stable dispersion where particles are distributed in a narrow size region (monodisperse distribution) is needed. Cohen *et al.* [33] presented a protocol for the production of stable monodisperse dispersions for *in vitro* studies. However, this may not mimic workers’ exposure to ENM powders where agglomerates are distributed into a wide size range, as seen here or as shown for example by Koivisto *et al.* [16]. Breaking the agglomerates for *in vitro* studies may also change the toxicological outcome as shown by Haniu *et al.* [59], who observed a decrease in toxicity when agglomerate size was decreased, obviously due to reduced exposure of the cells.

Demokritu *et al.* [60] made a direct comparison between *in vitro* and *in vivo* studies with physiologically equivalent doses and found that *in vitro* studies may not predict well toxicological outcomes *in vivo*. On the other hand, Khatri *et al.* [61] successfully linked *in vitro* experiments with human volunteers [62] and experimental animal [63] inhalation studies. This encourages developing *in vitro* studies so that the preliminary hazard assessment could be made for specific toxicological end points and for certain ENMs, as discussed more detailed in the Supplementary Information.

### 4.1. Comparison of ND Exposure with Provisional Exposure Limit Values for Nanoparticles and Diesel Particulate Matter

There are provisional exposure limit values for ENMs, called nano reference values (NRVs), which are designed to provide warning concentration levels for ENMs, to judge when exposure control measures should be taken [64,65]. The NRVs are designed for ENM particles in the range of 1–100 nm and thus cannot be utilized here, because the NDs had agglomerated into particles approximately over 500 nm. Thus, other limit values need to be considered in order to estimate the need for risk control measures.

For diesel particulate matter, 5 μg·m^−3^ is one of the proposed exposure limits to which humans may be exposed throughout their lifetime without experiencing any adverse noncancer health effects [66]. If one assumes that NDs and diesel particulate matter are similar in terms of toxicity, then this may be used to estimate an exposure limit when control measures are needed. ND concentrations were the highest (4.96 μg·m^−3^) during **WT2**. This suggests that risk control measures should be taken if sieving is performed extensively in a room. However, if the sieving is performed inside the fume chamber, as during **WT1**, ND exposure is reduced approximately to 5% of **WT2** ND exposure (Table 1).

The ND emission rates may be used to estimate the increase in ND concentration level if the work is scaled-up and if the work station environmental conditions remain similar [67] and in risk assessment tools, such as, Stoffenmanager Nano [68].

### 4.2. Comparison of a Work Day ND Dose with a Daily Dose of Submicrometer Urban Background Particles

Here the daily dose of ND agglomerates was compared with the daily dose of submicron urban background particles where exposures to local sources have been excluded. For an office worker with a typical activity pattern and who is living and working in a similar area where the workplace was located in this study, such modeled dose would be approximately 38 μg per work day [69]. The workers’ calculated dose of NDs during the measurement day was 74 ng which is approximately 500 times smaller than the daily dose of submicrometer urban background particles. Recently, the International Agency for Research on Cancer (IARC) classified diesel engine exhaust particles [70] and outdoor air pollution [71] as carcinogenic to humans (Group 1). NDs have not been classified for carcinogenicity but they have been assumed to be less toxic than other carbon particles [72,73]. This may be because diesel engine exhaust particles contain mutagenic and carcinogenic chemicals [66] which do not exist in NDs [34].

## 5. Conclusions

Risk assessment of inhalation exposure to airborne engineered nanomaterials is currently challenging due to lack of occupational exposure limits for ENMs. Here assessment of risk range was performed to nanodiamond (ND) inhalation exposure in a laboratory environment.

In *in vitro* studies, NDs showed low cytotoxicity, a clear production of ROS and a dose-dependent secretion of the pro-inflammatory cytokine, IL-1β, in THP-1 cells in THP-1 cells. Results suggested that ND exposure may cause a risk for activation of inflammatory cascade. The assessment of *in vitro* delivered dose on the cells was not reliable because the model used did not take into account ND agglomeration in the cell culture dispersion, and thus, risk range characterization based on *in vitro* dose-response was only discussed.

Because NDs were agglomerated to over 500 nm particles, mass concentration was the only reliable metric to discriminate the ND concentrations from background particles. During ND handling, sieving, and cleaning, ND exposure levels varied from 0.24 to 4.96 µg·m^−3^ (0.08 to 0.74 cm^−3^) while the work station air background concentration varied from 4.28 to 4.25 µg·m^−3^ (13 × 10^3^ to 5.9 × 10^3^ cm^−3^), respectively. Calculated dose of NDs during the work day was 0.02% of the average daily (24 h) dose of submicrometer urban background particles. The ND exposure levels were below 5 μg·m^−3^, which is one of the proposed exposure limits for diesel particulate matter.

Our results show that ND exposure levels were low, but ND hazard assessment was not sufficient in this study considering risk characterization and control. NDs short and long term health effects and translocation potential to other organs should be studied with *in vivo* methods. We recommend performing sieving in a fume chamber, which reduced exposure to 5% as compared with sieving without the cabinet, and use of respirators if there is potential exposure to high ND concentrations.

## Appendix

### Metrics to Characterize ND Concentrations

Averaged work task and the work station air background concentration ratios were used to identify the metrics that were sensitive to the ND concentrations compared to background concentrations (Table A1). A value less than 1 indicated that there was a particle sink, whereas a value over 1 was an indication of particle emissions. Because no significant particle sinks related to the work activity were identified (such as, for example, air cleaners or significant changes in coagulation sink), the values below 1 indicated uncertainties in determining the background concentration. This could be caused by fluctuations in incoming ventilation air concentration, unknown indoor sources or sinks, or changes in instrument response while measuring the concentrations. Here the incoming ventilation air particle number concentration (Figure 3a) and active surface area concentration (data not shown) varied markedly which explained the below 1 concentration ratios respective units.

**Table A1:** Concentration ratios for **WT1**, **WT2**, and **WT3** defined from particle number, current, and mass concentrations.

Metric Ratio	Instrument	WT1/BG1	WT2/BG2	WT3/BG2
Number ratio	SMPS **^a^**	0.90	0.93	0.92
	SMPS + OPC	1.11	1.16	0.87
	ELPI	1.11	1.14	0.83
	CPC	1.18	1.16	0.80
	DISCmini	0.85	1.22	0.93
Current ratio	DISCmini **^b^**	0.914	1.10	0.92
	ELPI **^c^**	1.02	1.17	0.96
Mass ratio	ELPI	1.14	1.32	0.62
	SMPS + OPC	1.04	2.29	1.41

Notes: **^a^** Background defined from incoming ventilation air measured over the corresponding work task; **^b^** Total current, *i.e.*, the sum of diffusion stage and filter stage currents; **^c^** The sum of currents measured by the electrometres from the impactor stages.

The incoming ventilation air total number concentration measured by the SMPS was approximately 8% higher than the work station number concentration (Figure 3a, Table A1). This means that work station total particle number concentration was derived by outdoor particles entering via ventilation inlets. The particle number concentration ratios shown in Table A1 revealed that ND concentrations could not be easily distinguished from the background particles if one used only the number concentration (designated by particle diameter *Dp.*). During **WT2**, the DISCmini number concentration ratio was elevated to 1.22, suggesting that ND exposure did occur. Figure 3b shows that the DISCmini predicted well the average particle size when there were no particles approximately over 1 μm present.

The ND concentrations did not increase the current levels (designated by particle diameter from *D_p_*^1^ to *D_p_*^2^) significantly during the work tasks (Table A1). Thus, their active surface area concentration was low as compared to the active surface area of the background particles.

During **WT2** and **WT3**, the mass concentration ratios (designated by particle diameter *D_p_*^3^) derived from the SMPS + OPC measurements were elevated to 2.29 and 1.41, respectively. The ELPI mass concentration ratios were considered unreliable (Table A1). This was most likely due to the low concentration of large particles in general and thus the signal to noise ratio of the electrometers was high.

Figure 4 shows that during the work tasks over 500 nm particle concentrations were leveled up which caused peaks in mass concentration (Figure 3b). These peaks were linked to individual work events (event times are not shown here), such as assembling the sieve or transport of NDs from glove box to the vibratory sieve shaker. Examination of electron micrographs revealed that these particles were agglomerated NDs (Figure 5). Sensitivity tests showed that the limit of 500 nm for the smallest size of ND agglomerates was very sensitive during the ND exposure in terms of number concentration but was negligible on mass concentration. For example, if the smallest size of NDs had been increased to 800 nm, this would have decreased mass concentration by 0.3%. Thus, the ND emissions from the processes affected mainly mass concentration but their contribution to total number concentration was insignificant (Figure 3a, Table A1); the most reliable metrics to characterize ND concentrations was mass (or volume) concentration.

## Supplementary Files

Supplementary File 1 Supplementary Information (PDF, 426 KB)
