# Supplementary material for: Range-Finding Risk Assessment of Inhalation Exposure to Nanodiamonds in a Laboratory Environment
Source: Int J Environ Res Public Health. 2014 May 16;11(5):5382–402. doi: 10.3390/ijerph110505382 (PMC4053885; doi:10.3390/ijerph110505382)
Supplement: Supplementary File 1 — Supplementary Information (PDF, 426 KB) [file ijerph-11-05382-s001.pdf]

Supplementary Information

Range-Finding Risk Assessment of Inhalation Exposure to Nanodiamonds in a Laboratory Environment

1. Methods

1.1. Calculation of Delivered Dose in in vitro-experiments

Delivered dose was calculated by using the *In vitro* Sedimentation, Diffusion and Dosimetry (ISDD) model developed by Hinderliter *et al.* [1]. The ISDD model is based on the general dynamics of particles in viscous media where mass transfer is driven by diffusion and sedimentation [2–4]. Because environmental conditions in *in vitro*—experiments are homogenous, advection is assumed to be negligible. The model is designed for primary particles and agglomerates which do not interact with each other, and thus the model does not take into account agglomeration during the simulation. For fractal particles, the model assumes that agglomerate pores are uniformly filled by the media and there are no flows through the agglomerate. Agglomerate behavior in viscous media in the ISDD model is described by Sterling *et al.* [5]. The ISDD model to calculate delivered dose of agglomerated particles requires the following parameters: temperature T (K), media density (g cm^{-3}) and viscosity μ (Pa s), media height h (m), ND hydrodynamic agglomerate size in the test media d_H (m), ND primary particle size d (m) and density ρ_p (g cm^{-3}), packing factor PF (–) and fractal dimension DF (–).

1.2. The ISDD Model Parameterization

Table S1 shows the differences between the *in vitro* experiments. In the ISDD model the temperature was 310 K, and we assumed that the media viscosity was 0.00069 Pa s and density 1 g cm^{-3} in both series.

Table S1. Parameters of the *in vitro*-experiments.

Parameter	Cell Death and Cytokine Secretion	ROS Generating Ability
Exposure time	6 h	3 h
Surface area of the culture dish	3.15 cm^2	1.72 cm^2
Cell culture medium	RPMI 1,640 with supplemental 1% PEST and 1% L-glutamine	dPBS without $CaCl_2$/$MgCl_2$
Volume of the ND dispersion	0.5 cm^3	0.3 cm^3

ND hydrodynamic diameter (d_H) was measured in RPMI 1,640 cell culture medium with supplemental 1% PEST and 1% L-glutamine by dynamic light scattering (DLS; Zetasizer Nano-ZS, Malvern Instruments, Malvern Worcs, UK) from a ND dispersion prepared in the same way as in the *in vitro* experiments. To meet the quality criteria required for the DLS measurements, the dispersion was filtered with 3.1 µm Chromacol 30-SF-31 30 MM GMF syringe filter (Thermo Scientific, Rockwood, TN, USA). The DLS measurements were performed using the following setting: scattering angle 173°, temperature 37 °C, and viscosity 0.00069 Pa s; sample equilibration time was 120 s, and the number of

runs was automatically set between 10 and 100. The average hydrodynamic diameter was obtained from seven successive measurements.

We assumed that the ND agglomerate packing factor was 0.637. Fractal dimension was solved from definitions of agglomerate porosity:

$$\varepsilon_{agg} = 1 - \left(\frac{d_{agg}}{d}\right)^{DF-3} \quad (1)$$

where d_{agg} is the agglomerate diameter which is assumed to be the same as the measured hydrodynamic diameter d_H, d is the primary particle size 5.5 nm (Figure S1), and the agglomerate density is [5]:

$$\rho_{agg} = \left(1 - \varepsilon_{agg}\right)\rho_p + \varepsilon_{agg}\rho_f \quad (2)$$

where ρ_{agg} is agglomerate density 0.5 g cm^{-3}, ρ_p is the primary particle density 3.15 g cm^{-3} (Figure S1), and ρ_f is the media density. If $\rho_f \ll \rho_{agg}$ it can be assumed that $\rho_f = 0$, which gives a solution for fractal dimension:

$$DF = \log_{\left(\frac{d_{agg}}{d}\right)}\left[\left(\frac{d_{agg}}{d}\right)^3\left(1 - \varepsilon_{agg}\right)\right] \quad (3)$$

Figure S1. Material data sheet.

Datasheet
Web 10-08-2012

uDiamond® Molto

High quality nanodiamond powder

Carbodeon uDiamond® Molto specific characteristics

Nanodiamond crystal size	4 - 6 nm
Nanodiamond content	≥ 97 wt.%
Oxidisable carbon content	≤ 2.5 wt.%
Metallic incombustible impurity content	≤ 1.2 wt.%
Bulk density	~ 0.5 g/cm^3
Pycnometric density	~ 3.1 – 3.2 g/cm^3
Specific surface area	~ 330 m^2/g
Crystal lattice constant	0.3573 ± 0.0005 nm
Graphitization in vacuum, starting at	~ 1100 °C
Oxidation in air, starting at	~ 450 °C
Moisture content	~ 4%

Main application areas include

- Thermal management technologies
- Polishing fluids and pastes
- Oil and lubricant additives
- Abrasive applications
- Polymer-diamond composites (predominantly with thermoplasts)
- Cold-plasma metal-diamond and polymer-diamond coatings
- Compacts and sintered pieces

From Carbodeon's online shop, product is supplied for R&D purposes only.

Carbodeon
SUPERHARD NANOMATERIALS

Carbodeon Ltd Oy
Pakkalankuja 5
FI-01510 Vantaa, Finland

Tel. +358 50 5678 828
www.carbodeon.com

1.3. Dose Translation from in vitro to Humans

A simple way to estimate human equivalent dose from *in vitro* dose is by normalizing the tissue areal dose with the internal surface area of the human lung. In this study, the culture dish surface area was either 3.15 or 1.72 cm^2 (Table S1) whereas human lung surface area is 6.33×10^5 cm^2 [6]. This gives conversion factors of 165×10^3 and 370×10^3 for the 3.15 and 1.72 cm^2 dishes, respectively, which were used to convert the *in vitro* dose to human equivalent dose.

2. Results

Depending on the nominal concentration of the ND dispersion, the ND agglomerate hydrodynamic size varied from 1.09 to 4.40 μm (Table S2). Repeated measurements showed an increasing trend in hydrodynamic diameter at nominal concentrations of 100 and 500 μg cm^{-3} (data not shown) which was most likely caused by agglomeration of NDs in the dispersion. Table S2 shows the delivered dose onto the cells solved with the ISDD model. The results are not reliable, because the dispersion was not stable and the ISDD model calculated delivered dose by means which cannot be applied for polydisperse particle dispersions such as used here (Table S2).

Table S2. Averages of hydrodynamic diameter (determined by dynamic light scatter) and corresponding polydispersivity index for 7 measurements (± shows the standard deviation), calculated fractal dimension, and delivered dose for cell death and cytokine secretion (ELISA) and ROS generating ability (ROS) experiments.

Nominal Concentration of ND Dispersion, (μg cm^{-3})	d_H, (μm)	Polydispersivity Index	*DF*	Delivered Dose, (μg)	
				ELISA	ROS
1	1.09 ± 0.35	0.67 ± 0.16	2.65	0.43	-
10	1.16 ± 0.08	0.37 ± 0.04	2.66	4.7	1.1
100	2.75 ± 0.25	0.18 ± 0.05	2.70	50	30
500	4.40 ± 0.95	0.32 ± 0.08	2.73	250	150

3. Discussion

In this study, we examined the effects of NDs with *in vitro* experiments using THP-1 cells and then we tried to assess if the *in vitro* delivered (settled) dose-responses could be translated for humans. A similar study was carried out by Khatri *et al.* [7] who studied printer emission particle dose-responses for inflammation, oxidative stress, genotoxic effects, and apoptosis in THP-1 human monocytic leukaemia cells. The *in vitro* results were found to be consistent with inhalation studies in human volunteers [8] and experimental animals [9]. This risk assessment method is straightforward and relatively easy to perform, but can it really be used as a preliminary occupational hygienic risk assessment tool? In the following, we briefly discuss weaknesses and limitations in this approach.

In regulatory risk assessment of chemicals, *in vitro* assays are currently only used for genotoxicity assessment. These studies are primarily applied for qualitative hazard identification (hazard present/ not present), and dose-response data are not routinely extrapolated to the situation *in vivo*. The use of genotoxicity tests in hazard identification is based on the idea that genotoxicants that primarily target DNA should be able to do so both *in vitro* and *in vivo*. Validation of the *in vitro* genotoxicity assays has

shown that this can be assumed with reasonable accuracy. Yet, confirmation for positive *in vitro* findings on genotoxicity is usually sought from genotoxicity assays *in vivo*. Similar use of *in vitro* inflammatory assays in hazard identification appears very promising, but is presently still in an exploratory phase and does not yet have a regulatory role. Cytotoxicity assessment is integral in dose-range finding of *in vitro* testing for genotoxicity and immunotoxicity, but regulatory assessment of general toxicity is still exclusively performed *in vivo*.

Regulatory risk assessment of chemicals, which forms the basis for setting of occupational exposure limits, relies on rather extensive *in vivo* data covering various types of adverse effects in different target organs. If an *in vitro* model were to be used as a preliminary tool to assess new materials for such effects, several reservations should be made. Obviously, extrapolations to the *in vivo* situation can only concern those effects that are studied *in vitro*. Conclusions cannot be drawn on effects that are not associated with the endpoints examined. For instance, an *in vitro* assay for inflammatory potential will not necessary tell anything about genotoxic potential, and *vice versa*. The cell type applied in the *in vitro* experiments may be more or less representative of similar tissue or cells *in vivo*, but may not be a proper model for other types of cells and tissues. ENM uptake and processing may largely vary among cells of different origin. *In vivo*, toxic effects often require the interplay of different cell types and tissues—a situation that is difficult to reproduce *in vitro*. Translocation of ENMs to other organs can occur only *in vivo*. It is possible that the mechanisms that bring out toxic effects *in vivo* do not exist or are only partly present *in vitro*.

Another critical issue concerns differences in exposure between *in vitro* and *in vivo*. *In vitro*, ENM exposures are mostly performed as dispersions, whereas occupational exposures normally occur through inhalation of aerosols. While *in vitro* experiments typically involve short-term and high-dose exposures, humans are ordinarily exposed to low doses repeatedly during a long period of time. Although most concern in human exposure is in long-term effects, the *in vitro* approaches have been suggested to be used primarily for modelling short-term effects. The *in vitro* models are based on a number of assumptions some of which may not be well justified. For instance, despite this obvious differences between *in vitro* and *in vivo* exposure, cellular exposure to particles is assumed to remain the same, lung clearance is considered insignificant, and particle coating with biomolecules is not expected to influence the dose-response [10,11]. In reality, exposure route, dose and dose rate, and bio-corona may strongly influence the type and level of toxic effects encountered, and different toxic mechanisms may be operative at different exposure regimes.

It is evident from what is discussed above that quantitative risk assessment of workplace ENM exposure using an *in vitro* model is a complex question. Considering conventional health risk assessment of chemicals, such approaches may seem superficial and trivial. Naturally, the idea is not to replace thorough risk assessment with a simple *in vitro* assay, but to search for a rapid tool for the first identification of hazardous ENMs in a scheme where a multitude of variable ENMs without toxicological information are increasingly used at workplaces. The possible success of such an exercise depends on a number of issues. It cannot be expected to cover all aspects of toxicity but should most probably be limited to a specific question that would ideally be critical for ENM toxicity. Ability to evoke persistent inflammation at a low dose might be one such phenomenon. A crucial question is whether the endpoints studied and the *in vitro* models used are able to reveal this

effect. An answer can only be obtained by validation studies that would systematically compare responses *in vitro* and *in vivo*.

References

1. Hinderliter, P.M.; Minard, K.R.; Orr, G.; Chrisler, W.B.; Thrall, B.D.; Pounds, J.G.; Teeguarden, J.G. ISDD: A computational model of particle sedimentation, diffusion and target cell dosimetry for *in vitro* toxicity studies. *Part. Fibre Toxicol.* **2010**, *7*, doi:10.1186/1743-8977-7-36
2. Elimelech, M.; Gregory, J.; Jia, X.; Williams, R. *Particle Deposition and Aggregation—Measurement, Modelling and Simulation*; Elsevier: Woburn, MA, USA, 1995.
3. Dusenberry, D.B. *Living at the Micro Scale: The Unexpected Physics of Being Small*; Harvard University Press: Cambridge, MA, USA, 2009.
4. Bird, R.B.; Stewart, W.E.; Lightfoot, E.N. *Transport Phenomena*; John Wiley & Sons, Inc.: Danvers, MA, USA, 1960.
5. Sterling, M.C.; Bonner, J.S.; Ernest, A.N.; Page, C.A.; Autenrieth, R.L. Application of fractal flocculation and vertical transport model to aquatic sol-sediment systems. *Water Res.* **2005**, *39*, 1818–1830.
6. Xu, G.B.; Yu, C.P. Deposition of diesel exhaust particles in mammalian lungs a comparison between rodents and man. *Aerosol Sci. Technol.* **1987**, *7*, 117–123.
7. Khatri, M.; Bello, D.; Pal, A.K.; Cohen, J.M.; Woskie, S.; Gassert, T.; Lan, J.; Gu, A.Z.; Demokritou, P.; Gaines, P. Evaluation of cytotoxic, genotoxic and inflammatory responses of nanoparticles from photocopiers in three human cell lines. *Part. Fibre Toxicol.* **2013**, *10*, doi:10.1186/1743-8977-10-42.
8. Khatri, M.; Bello, D.; Gaines, P.; Martin, J.; Pal, A.; Gore, R.; Woskie, S. Nanoparticles from photocopiers induce oxidative stress and upper respiratory tract inflammation in healthy volunteers. *Nanotoxicology* **2013**, *7*, 1014–1027.
9. Pirella, S.; Molina, R.; Watson, C.; Cohen, J.; Bello, D.; Demokritou, P.; Brain, J. Effects of copy center particles on the lungs: A toxicological characterization using a Balb/c mice model. *Inhal. Toxicol.* **2013**, *25*, 498–508.
10. Demokritou, P.; Gass, S.; Pyrgiotakis, G.; Cohen, J.M.; Goldsmith, W.; McKinney, W.; Frazer, D.; Ma, J.; Schwegler-Berry, D.; Brain, J.; *et al*. An *in vivo* and *in vitro* toxicological characterisation of realistic nanoscale CeO_2 inhalation exposures. *Nanotoxicology* **2013**, *7*, 1338–1350.
11. Cohen, J.; DeLoid, G.; Pyrgiotakis, G.; Demokritou, P. Interactions of engineered nanomaterials in physiological media and implications for *in vitro* dosimetry. *Nanotoxicology* **2013**, *7*, 417–431.

© 2014 by the authors; licensee MDPI, Basel, Switzerland. This article is an open access article distributed under the terms and conditions of the Creative Commons Attribution license (http://creativecommons.org/licenses/by/3.0/).
